# Statistical Modeling of Indirect Paths for UWB Sensors in an Indoor Environment

**DOI:** 10.3390/s17010043

**Published:** 2016-12-27

**Authors:** Moona Lee, Joon-Yong Lee

**Affiliations:** 1 Siemens Healthcare Ltd., Pohang-si 37668, Korea; lma1230@naver.com; 2 School of Computer Science & Electrical Engineering, Handong Global University, Pohang-si 37554, Korea

**Keywords:** ultra-wideband, indirect path, human tracking

## Abstract

In this paper, we present a statistical model of an indirect path generated in an ultra-wideband (UWB) human tracking scenario. When performing moving target detection, an indirect path signal can generate ghost targets that may cause a false alarm. For this purpose, we performed radar measurements in an indoor environment and established a statistical model of an indirect path based on the measurement data. The proposed model takes the form of a modified Saleh–Valenzuela model, which is used in a UWB channel model. An application example of the proposed model for mitigating false alarms is also presented.

## 1. Introduction

Recently, accurate passive localization has become a very important technology for the purposes of security, intrusion detection, and robot tracking, to name a few. When a radar sensor is used, a method to detect a moving target by sensing a change in the received signals has been widely used. In particular, ultra-wideband (UWB) radars are more advantageous than the existing Doppler radars since they can accurately detect even tiny movements of a target. However, in an indoor environment, a variety of problems may be caused because of a dense multipath. Bartoletti et al. [1,2] showed that the localization accuracy can be improved by appropriate allocation of resources and selection of observations in an indoor environment with multipath, clutter, and line-of-sight blockage. A blind zone, an area where the target cannot be detected, can cause various problems. Sobhani et al. [3] proposed a modified particle filter to resolve the tracking issues caused by blind zones.

**Figure 1:**
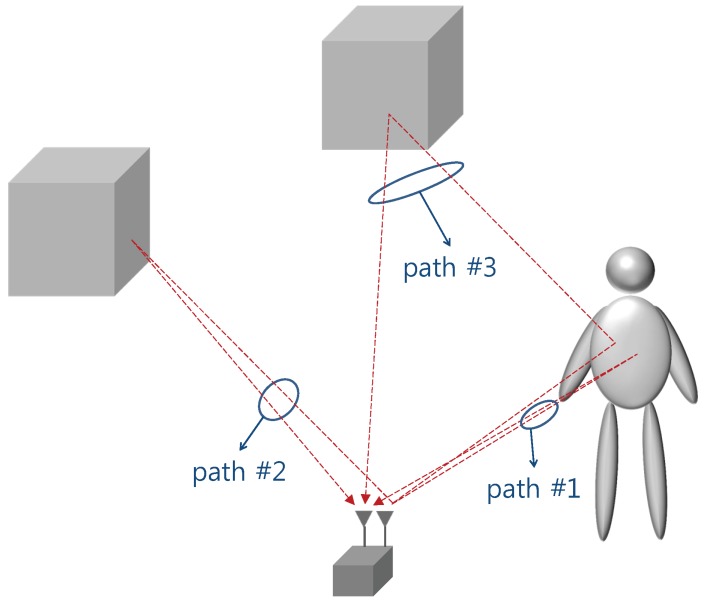
Different reflection scenarios caused by a moving human.

An indirect reflection that includes the target and other objects in the reflection path can cause a ghost problem [4,5,6]. Figure 1 illustrates this phenomenon. In this figure, path number 1 (direct path) contains the range information of the target. Path number 2 receives static background signals that can be removed via the moving target indication (MTI). Path number 3, namely the indirect path, contains multiple reflections from a human body and the background objects. The indirect path signals survive the MTI process and thus generate ghost targets. The deterministic method has been suggested for detecting an indirect path. For example, Shen and Molisch [4] adopted a deterministic approach, using measurement parameters such as the time of arrival (ToA), direction of departure, and direction of arrival. If a statistical model of the indirect path is available, it will be possible to employ a stochastic approach based on the model. However, no statistical model of the indirect path has yet been proposed, although a study on propagation characterization for UWB sensors exists. In this study, we performed statistical modeling of the indirect path that occurs under an indoor human tracking condition. To achieve this, a comprehensive measurement campaign was performed in an indoor environment, and a cluster model was established based on the measured data. This model can be applied to reject ghost targets that are generated by an indirect path.

The rest of this paper is organized as follows: In Section 2, the measurement campaign to collect the radar scan data is described. In Section 3, the indirect path model that is established based on these data is introduced. Section 4 introduces an indirect path model application example of false alarm mitigation for one-dimensional (1D) two-target tracking.

## 2. Measurement Campaign

Radar measurements were conducted for the statistical modeling of an indirect path that occurred during moving target detection in indoor environments. More specifically, we modeled the arrival time and the strength of the indirect path. These parameters can vary depending on factors such as the geometric shape and the reflection coefficient of a target. In this study, we assume that the target is a human. Experiments were conducted in buildings inside Handong University, and 2293 scans were obtained. Table 1 summarizes the environment in which the measurements were taken. The radar used in the experiment is the PulsOn 400 monostatic radar kit manufactured by Time Domain, Inc. (Huntsville, AL, USA); it has two omni-directional dipole antennas attached with a passband of 3.1–5.3 GHz. A radar was installed at a height of 0.8 m, and the radar scans were collected when one person walked around the radar. Five scans were obtained per second, and the sampling time was 61 ps. We attempted to remove as many sources of non-stationary clutter, such as running fans and swaying curtains other than moving targets, as possible.

Signals without moving targets were also measured and used as reference for the MTI. The template waveforms of the radar signals can be approximated by [5]:

(1) $$s\left(\tau \right)=A\exp\left(-a\tau^{2}\right)\sin\left(b\tau \right),$$

where $a=5.55\times 10^{18}$, $b=26.15\times 10^{9}$, and *A* denotes a constant.

Let $r\left(\tau ;t\right)$ be the received signal with *τ* being the propagation delay (fast time) and *t* being the measurement time (slow time). Without loss of generality, $r_{\mathrm{ref}}\left(\tau \right)=r\left(\tau ;0\right)$ is selected as the reference signal for stationary clutter removal, and the difference signal $r_{\mathrm{dif}}\left(\tau ;t\right)$ is defined as:

(2) $$r_{\mathrm{dif}}\left(\tau ;t\right)=r\left(\tau ;t\right)-r_{\mathrm{ref}}\left(\tau \right).$$

**Figure 2:**
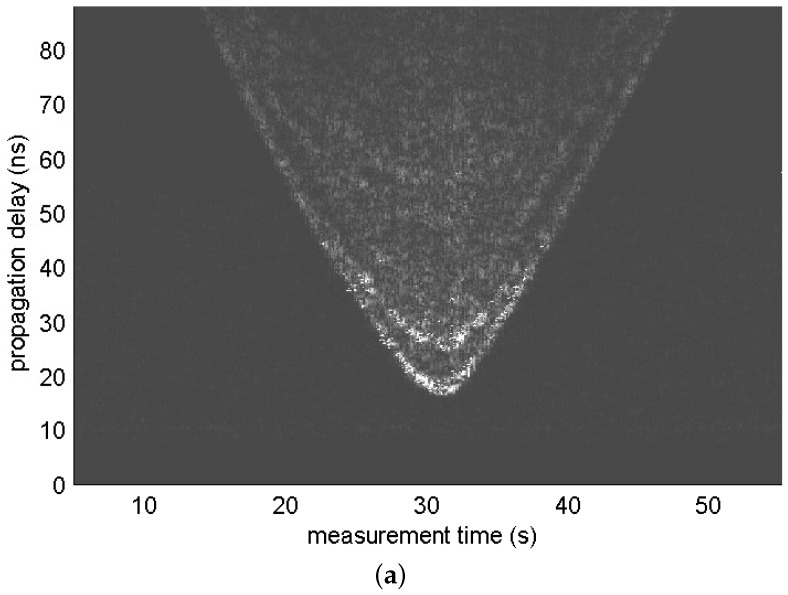

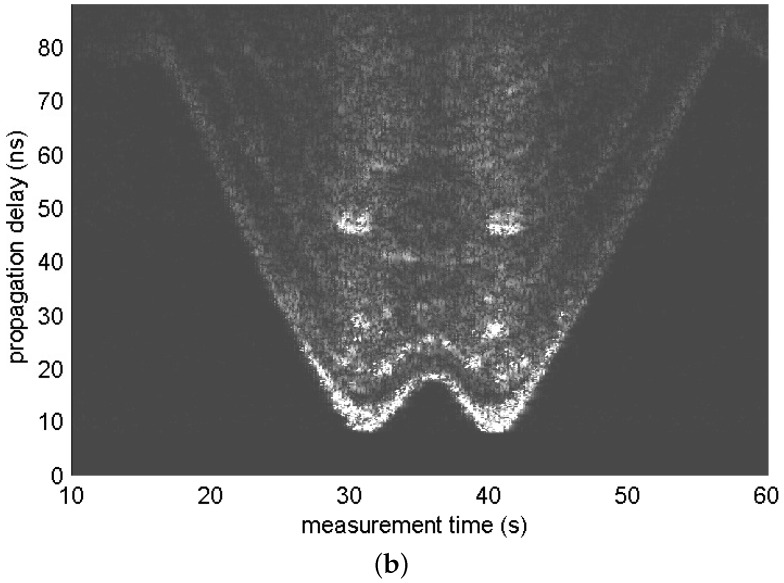
Radargrams of measurement sets (**a**) 6 and (**b**) 7.

Slow fluctuation of the power level with respect to time *t* at the obtained signal $r_{\mathrm{dif}}\left(\tau ;t\right)$ was observed, and high-pass filtering was done to remove it. The resulting signal is given by

(3) $$\begin{array}{c} z\left(\tau ;t\right)=\int_{-\infty }^{\infty }r_{\mathrm{dif}}\left(\tau ;t\right)g_{\mathrm{HP}}\left(t-\xi \right)d\xi , \end{array}$$

where $g_{\mathrm{HP}}\left(t\right)$ denotes the impulse response of the high-pass filter. Signal $z\left(\tau ;t\right)$ includes the indirect path signal components as well as a direct path signal. Figure 2 shows the radargrams of measurement sets 6 and 7. Signal components that arrived the earliest in each scan observed in the figure represent the trajectory of the distance between the radar and the moving human. We can observe other signal components that arrived later than the abovementioned signal components; these are the indirect path signal components.

## 3. Indirect Path Model

First, an impulse response was obtained by applying the CLEAN algorithm [7] to signal $z\left(\tau ;t\right)$ given by Equation (3). The iteration process was supposed to stop when either of the following conditions was met:

- Captured energy is greater than 90% of the total energy.
- Path strength is less than four times the noise standard deviation.

**Figure 3:**
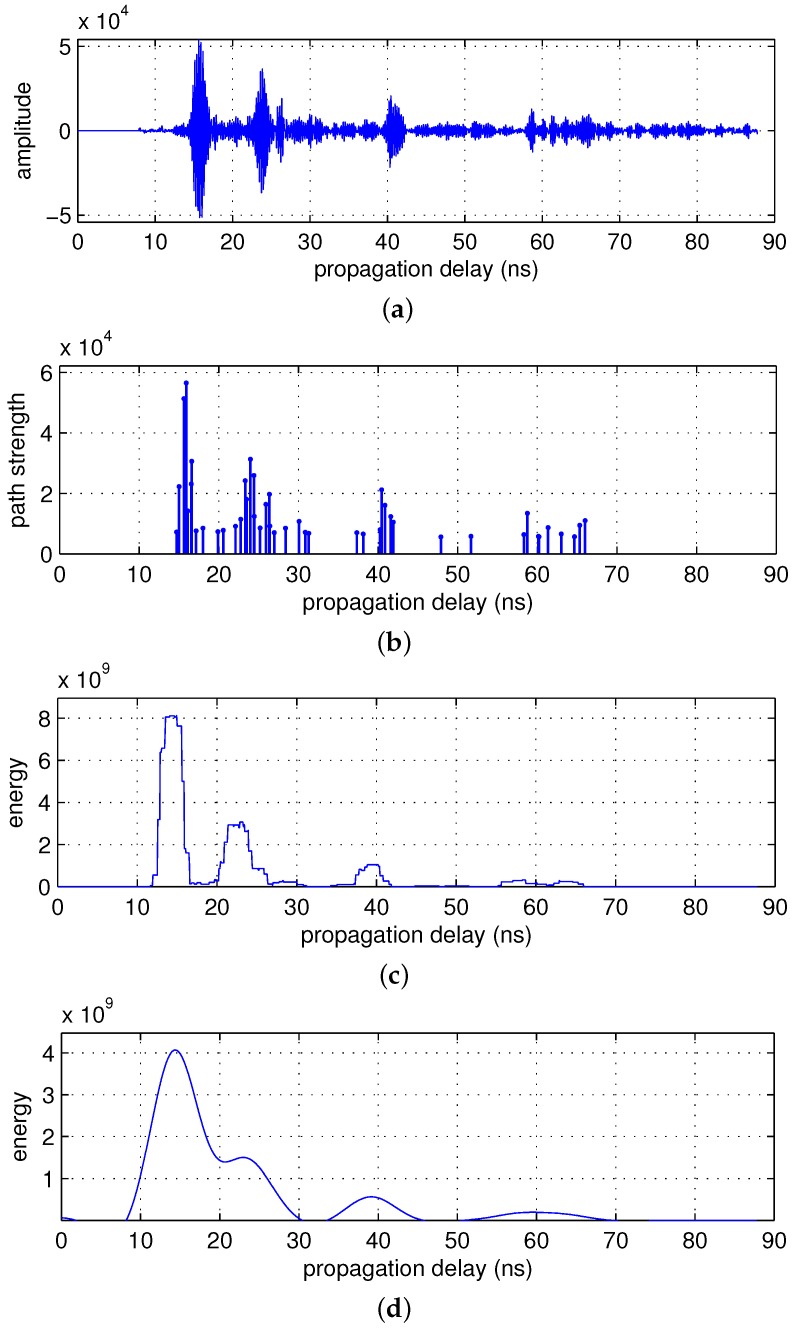
Deconvolution and cluster identification for the 170th scan of measurement set 7. (**a**) $z\left(\tau ;t\right)$; (**b**) magnitude of the impulse response; (**c**) output of a sliding correlator; and (**d**) output of a low-pass filter.

Figure 3a,b shows signal $z\left(\tau ;t\right)$ obtained from the 170th scan of measurement set 7 and the magnitude of its impulse response, respectively. Interestingly, we can observe that the signal components are clustered as in the UWB channel model. More than two clusters are observed, which indicates that there are clusters generated by indirect reflections, assuming that only one cluster is generated by a direct reflection. In this study, we use a cluster model with the following impulse response:

(4) $$h\left(\tau ;t\right)=\sum_{l=0}^{L(t)}\sum_{k=0}^{K(t)}\alpha_{k,l}\left(t\right)\delta_{D}\left(\tau -T_{l}\left(t\right)-\tau_{k,l}\left(t\right);t\right),$$

where $\delta_{D}\left(\cdot \right)$ denotes the Dirac delta function. The parameter $T_{l}\left(t\right)$ represents the delay of the $l^{\mathrm{th}}$ cluster, $\tau_{k,l}\left(t\right)$ indicates the delay of the ray relative to the cluster arrival time, and $\tau_{0,l}\left(t\right)=0$ for all *l*. The parameter $\alpha_{k,l}\left(t\right)$ denotes the path strength. The subscript {k,l} indicates that the quantities depend on the $l^{\mathrm{th}}$ cluster and the $k^{\mathrm{th}}$ ray; these parameters are dependent on the measurement time *t*. Assuming that the direct path signal always arrives earlier than the indirect path signals, $T_{0}\left(t\right)$ is the arrival time of a cluster generated by a direct reflection and $T_{l}\left(t\right)$ with l>0 is the arrival time of a cluster generated by an indirect reflection.

To identify a cluster from the impulse response, we first carried out a sliding correlation between the squared version of the impulse response and a 3-ns-wide rectangular window. Figure 3c illustrates this example. Although visual inspection is widely used for cluster identification, this has the disadvantage of a strong dependence on human intuition. Instead, we applied additional low-pass filtering to signals obtained through a sliding correlation (see Figure 3d) and performed clustering by finding a location where the slope of the tangent of the low-pass filter output exceeded the threshold, 1.

### 3.1. Path Arrivals

**Figure 4:**
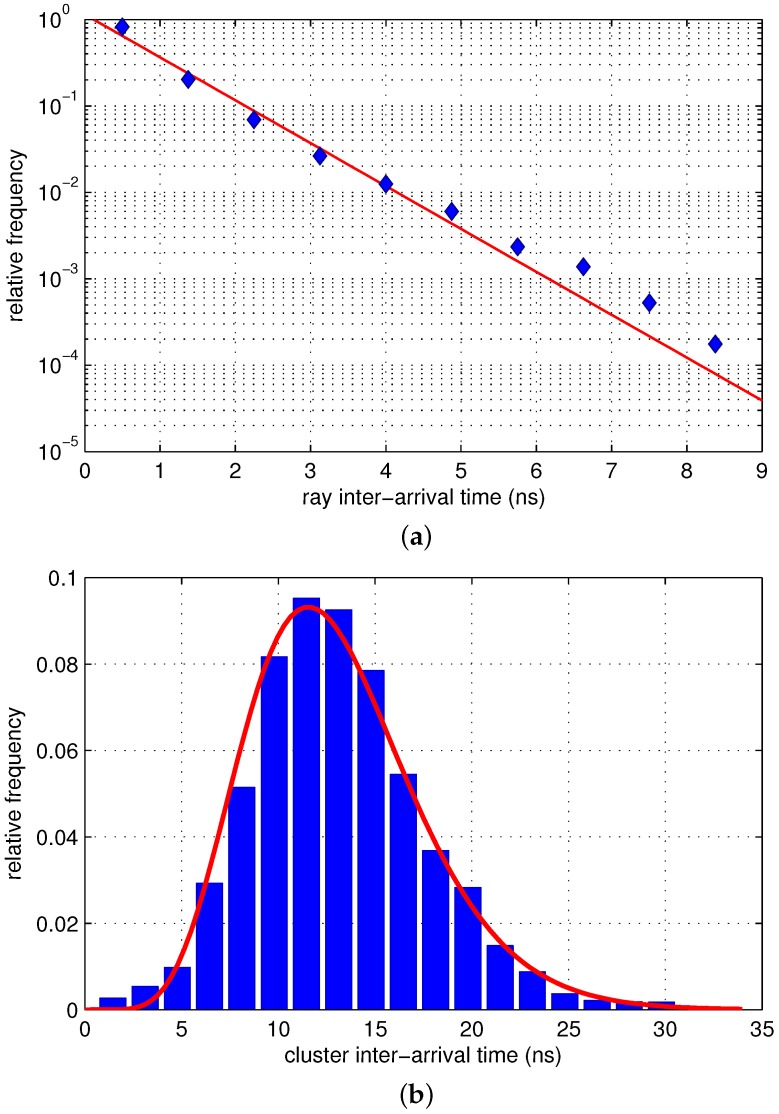
Distributions of the (**a**) ray and (**b**) cluster inter-arrival times.

Ray and cluster arrivals are modeled as Poisson processes in the modified Saleh–Valenzuela (S–V) model widely used as the UWB channel model. In this study, exponential fit was applied to the inter-arrival time of rays as in the modified S–V model. Figure 4a shows the distribution of the inter-arrival time of the rays and its exponential fit. The ray arrival rate, namely *λ*, was obtained to be 1.1521 by measuring the mean inter-ray arrival time.

In contrast to ray arrival, the inter-arrival time of clusters, namely *δ*, is closer to the following gamma model than the exponential model:

(5) $$f_{\delta }\left(\delta \right)=\frac{\delta^{K-1}e^{-\delta /\theta }}{\theta^{K}\Gamma \left(K\right)}.$$

Figure 4b shows the distribution of inter-arrival time of the clusters and its gamma fit with K=8.4476 and θ=1.5510. To examine the goodness of fitness, a chi-squared test was performed. The resulting $\chi^{2}$ value was 6.36, which was less than the critical value, 7.81, corresponding to a 5% significance level and three degrees of freedom.

### 3.2. Path Strengths

As in the modified S–V model [8], the path strength is assumed to follow log-normal fading, with its mean energy decreasing exponentially with excess delay, as follows:

(6) $$\begin{array}{c} E\left[{\left|\alpha_{k,l}\right|}^{2}\right]\propto e^{-T_{l}/\Gamma }e^{-\tau_{k,l}/\gamma }, \end{array}$$

where Γ and *γ* denote the cluster and ray decay factors, respectively. The process to find an optimal value of model parameters *γ* and Γ from the measured data is similar to the approach described in [9]. First, the ray decay factor *γ* is obtained according to the least squared error criterion:

(7) $$\begin{array}{c} \gamma =\arg\min_{\gamma^{\prime }}\left[\min_{\left\{\beta_{l}^{\left(m\right)}\right\}}\sum_{m}\sum_{l}\sum_{k}{\left[20\log_{10}\left|\alpha_{k,l}^{\left(m\right)}\right|-\beta_{l}^{\left(m\right)}+\frac{10\left(\tau_{k,l}^{\left(m\right)}-\tau_{0,l}^{\left(m\right)}\right)}{\gamma^{\prime }\ln10}\right]}^{2}\right], \end{array}$$

where *m* refers to an index of the used radar scan. Parameter $\beta_{l}^{\left(m\right)}$ accounts for the energy of the $l^{\mathrm{th}}$ cluster of the $m^{\mathrm{th}}$ radar scan in the decibel scale, which is chosen to minimize the squared error for a given value of $\gamma^{\prime }$. Once the value of *γ* is determined, an optimal value of the $l^{\mathrm{th}}$ cluster energy can be obtained as:

(8) $$\begin{array}{c} \nu_{l}^{\left(m\right)}=\arg\min_{\nu }\left[\sum_{k}{\left[20\log_{10}\left|\alpha_{k,l}^{\left(m\right)}\right|-\nu +\frac{10\left(\tau_{k,l}^{\left(m\right)}-\tau_{0,l}^{\left(m\right)}\right)}{\gamma \ln10}\right]}^{2}\right]. \end{array}$$

**Figure 5:**
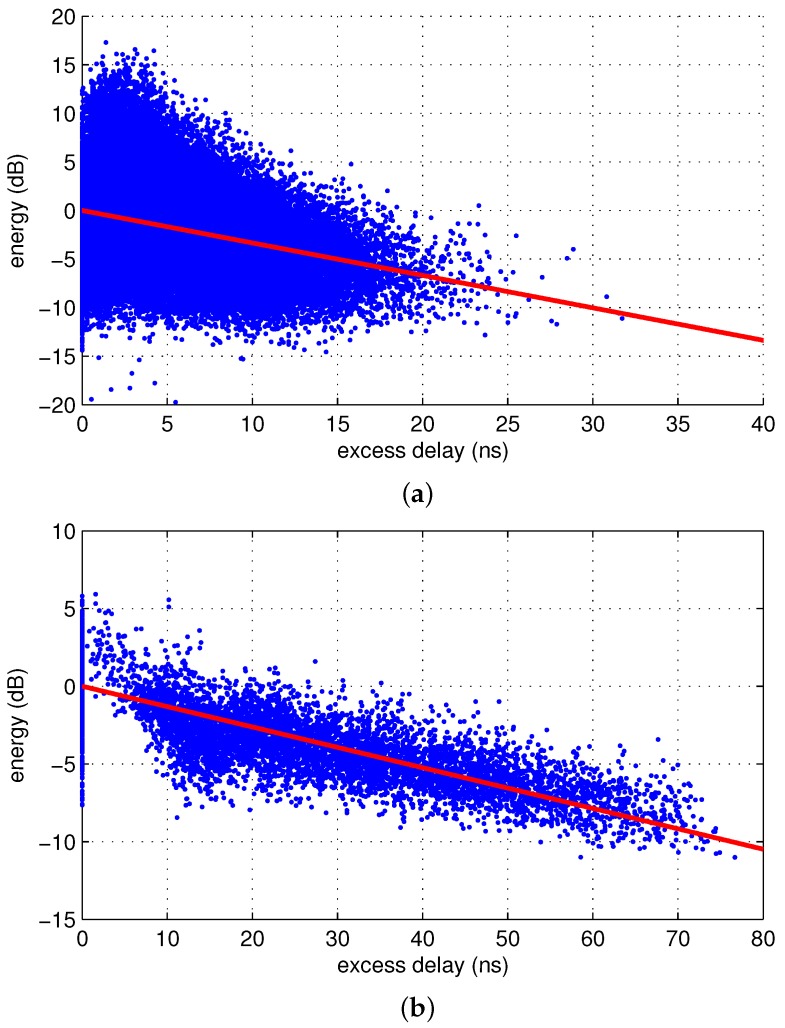
Scatter plots of the (**a**) ray and (**b**) cluster energies.

Now, the optimal value of Γ can be found by:

(9) $$\begin{array}{c} \Gamma =\arg\min_{\Gamma^{\prime }}\left[\min_{\left\{\phi^{\left(m\right)}\right\}}\sum_{m}\sum_{l}{\left[\nu_{l}^{\left(m\right)}-\phi^{\left(m\right)}+\frac{10\left(T_{l}^{\left(m\right)}-T_{0}^{\left(m\right)}\right)}{\Gamma^{\prime }\ln10}\right]}^{2}\right], \end{array}$$

where the cluster energies of the $m^{\mathrm{th}}$ signal are scaled by the constant $\phi^{\left(m\right)}$ such that the squared error is minimized. Figure 5 shows the scatter plots of the ray and cluster energies. The values of the fading parameters are listed in Table 2.

## 4. Application of the Indirect Path Model to Two-Target Tracking

### 4.1. Test Scenario

**Figure 6:**
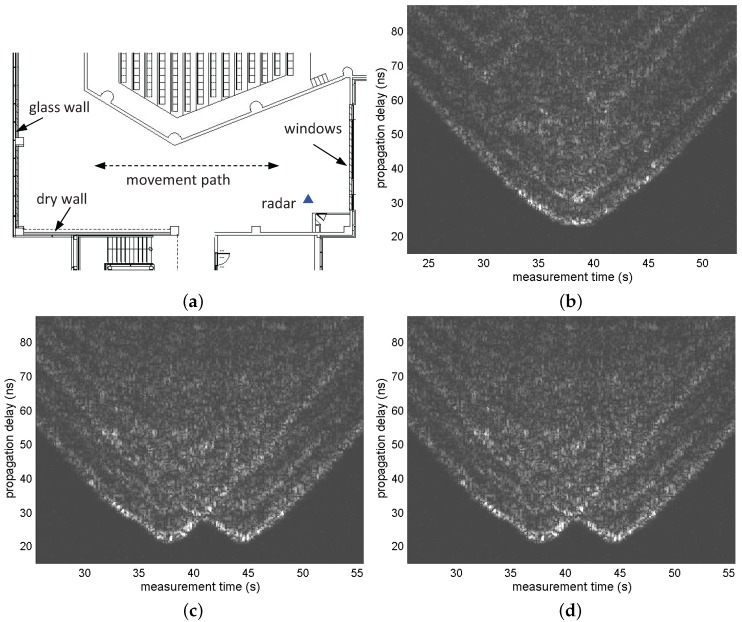
(**a**) Floor plan of the building where the experiments were conducted; (**b**) radargrams of Scenario 1. One target approached the radar and then moved away while the other repeated motions approaching the radar from a distance and returned back; (**c**) radargrams of Scenario 2. One target approached the radar and returned, and the other moved along the same path with a certain constant delay; and (**d**) radargrams of Scenario 3. Both targets approached the radar with some gap between them and moved away from the radar simultaneously.

This section presents an application example that applies the indirect path model introduced in Section 3 to 1D two-target tracking in an indoor environment. For this purpose, another set of radar measurements independent of the measurement data introduced in Section 2 was conducted. One monostatic radar was installed in an indoor environment, and two people moved along a straight line near the radar. Measurements were conducted for three test scenarios: Figure 6a shows the movement path of the two targets. The targets moved with different timings along the same path in all three scenarios. Figure 6b–d shows the radargrams of each scenario.

**Figure 7:**
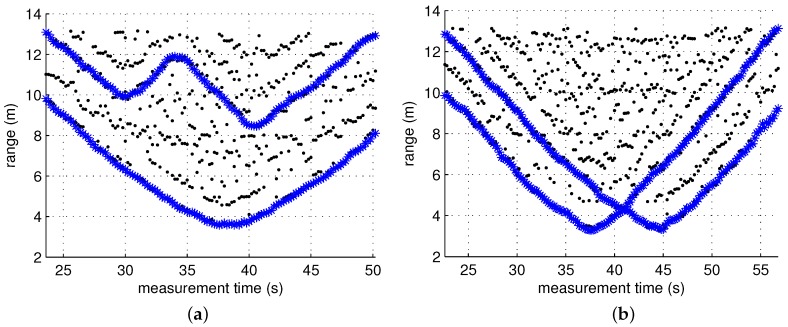

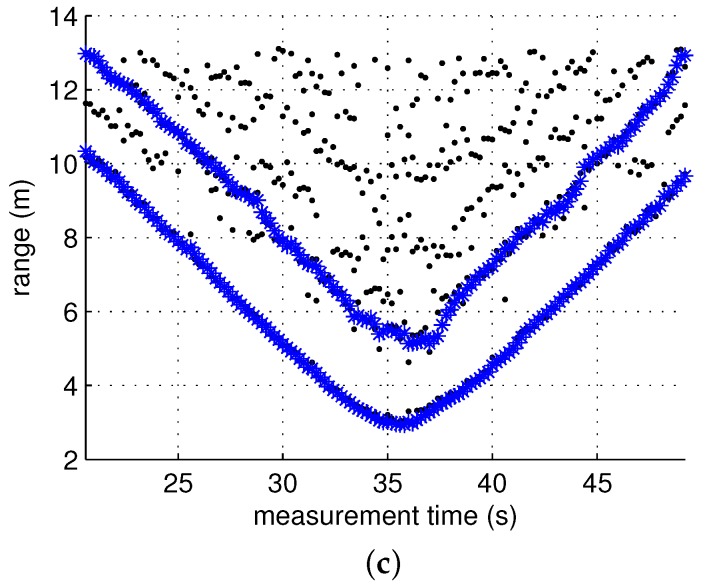
Scatter plots of clusters without ghost rejection for each scenario. (**a**) Scenario 1; (**b**) Scenario 2; and (**c**) Scenario 3.

The figures in Figure 7 are the scatter plots of the cluster arrivals obtained using the CLEAN algorithm and cluster identification. As shown in these figures, in most scans, more clusters than the number of targets were detected, which was attributed to indirect reflections. Among the ToAs of the detected clusters, the ToAs of the clusters generated by a direct reflection at each target contain the range information. Our task here is to reduce a false alarm rate by screening out clusters that are likely to have been generated by an indirect reflection among the many detected clusters. This task is performed on the basis of the cluster unit by assuming that the first arriving ray inside each cluster contains the range information of the target. This algorithm is applied on a single-scan basis. It is a type of screening process of observations detected in a single scan, which is conducted independently of the detection results of the other scans. Thus, it is carried out independently of other tracking tasks such as track association and prediction. This is to show that the proposed screening algorithm can achieve false alarm mitigation independently from most of the ghost rejection algorithm applied to the data association phase. Therefore, if tracking tasks such as track association and prediction are performed after the screening, it is expected to further enhance the performance.

### 4.2. Screening Algorithm

With ToAs and strengths of clusters obtained by applying the CLEAN algorithm and the clustering algorithm to the $m^{\mathrm{th}}$ scan, the following observation matrix can be built:

(10) $$R^{\left(m\right)}=\left[{\underline{T}}^{\left(m\right)},\;{\underline{\nu }}^{\left(m\right)}\right]=\left[\begin{array}{cc} T_{0}^{\left(m\right)}, & \nu_{0}^{\left(m\right)}\\ T_{1}^{\left(m\right)}, & \nu_{1}^{\left(m\right)}\\ \vdots & \vdots \\ T_{L_{m}-1}^{\left(m\right)}, & \nu_{L_{m}-1}^{\left(m\right)} \end{array}\right],$$

where column vectors ${\underline{T}}^{\left(m\right)}$ and ${\underline{\nu }}^{\left(m\right)}$ refer to the vectors of the arrival time and the strength of the detected clusters, respectively. Here, we assume $T_{0}^{\left(m\right)}<T_{1}^{\left(m\right)}<\cdots <T_{L_{m}-1}^{\left(m\right)}$. Parameter $L_{m}$ refers to the number of clusters, and $\nu_{l}^{\left(m\right)}$, which is an estimated strength of ${\left(l+1\right)}^{\mathrm{th}}$ cluster, is determined by Equation (8). The first observation vector $\left(T_{0}^{\left(m\right)},\nu_{0}^{\left(m\right)}\right)$ is assumed to be generated by a direct reflection at a target closer to the radar. For the sake of convenience, a target that is closer to the radar is indexed 0 and the other target is indexed 1. Note that this index is a value assigned only within a single scan. Which target is closer to the radar depends on the movements of targets; therefore, an index of the same target can also differ according to *m*. Let us also define an $L_{m}$-dimensional vector ${\underline{\mu }}^{\left(m\right)}$, which represents a particular association between targets and observations inside the $m^{\mathrm{th}}$ scan, as:

(11) $${\underline{\mu }}^{\left(m\right)}=\left[\mu_{0}^{\left(m\right)},\mu_{1}^{\left(m\right)},\cdots ,\mu_{L_{m}-1}^{\left(m\right)}\right].$$

Here, an all-zero vector or an all-one vector is not considered to be a candidate of ${\underline{\mu }}^{\left(m\right)}$. Then, the number of possible vectors ${\underline{\mu }}^{\left(m\right)}$ is $2^{L_{m}-1}-2$. The first “0” and the first “1” in the elements of vector ${\underline{\mu }}^{\left(m\right)}$ represent the direct reflections at target 0 and target 1, respectively, and the other elements indicate the indirect reflections. Since $\mu_{0}^{\left(m\right)}=0$ is satisfied for all *m*, our task is to determine the position of the first non-zero element in the vector ${\underline{\mu }}^{\left(m\right)}$, i.e., to find the first observation vector associated to target 1 from the matrix $R^{\left(m\right)}$.

Let $H_{j}^{\left(m\right)}$ denote the hypothesis that the first non-zero element of ${\underline{\mu }}^{\left(m\right)}$ is $\mu_{j}^{\left(m\right)}$. Then, we can define the following likelihood function to evaluate each hypothesis:

(12) $$L\left(\left.H_{j}^{\left(m\right)}\right|R^{\left(m\right)}\right)=\max_{{\underline{\mu }}^{\left(m\right)}\in A_{j}^{\left(m\right)}}f_{\left.\underline{T\nu }\right|\underline{\mu }}\left(\left.{\underline{T}}^{\left(m\right)},{\underline{\nu }}^{\left(m\right)}\right|{\underline{\mu }}^{\left(m\right)}\right),\;0\leq j\leq L_{m}-1,$$

where set $A_{j}^{\left(m\right)}$ is a set of vector ${\underline{\mu }}^{\left(m\right)}$s that satisfy $H_{j}^{\left(m\right)}$, in other words,

(13) $$A_{j}^{\left(m\right)}=\left\{{\underline{\mu }}^{\left(m\right)}\left|\arg\min_{l}\left[\mu_{l}^{\left(m\right)}\neq 0\right]=j\right.\right\}.$$

In Equation (12), the likelihood function is defined as the maximum of the conditional density with respect to all vectors $\left\{{\underline{\mu }}^{\left(m\right)}\right\}$ that belong to set $A_{j}^{\left(m\right)}$ because the distribution of ${\underline{\mu }}^{\left(m\right)}$ is unknown. For the conditional density shown in Equation (12), all parameters are independent and every observation vector is partitioned by ${\underline{\mu }}^{\left(m\right)}$; therefore, they can be easily evaluated. For more details, refer to the Appendix A.

The hypotheses ${\left\{H_{j}^{\left(m\right)}\right\}}_{j=0}^{L_{m}-1}$ are all mutually exclusive and exhaustive. In contrast to the hypothesis testing approach in which only one hypothesis is accepted, we are more interested in selecting a set of hypotheses that are decently probable. This is to utilize the selected candidates of the direct reflection for further tracking tasks such as track association and filtering. We choose $H_{j}^{\left(m\right)}$ if and only if $L\left(\left.H_{j}^{\left(m\right)}\right|R^{\left(m\right)}\right)$ is greater than the threshold $\theta_{L}$. Here, in order to define the probability of false alarm $\left(P_{\mathrm{FA}}\right)$ and the probability of detection $\left(P_{D}\right)$, we defined the discrete cells of the measurement time for the sake of convenience. The size of the time cell is 1.95 ns of the time resolution, and $P_{\mathrm{FA}}$ and $P_{D}$ are defined as follows:

$$\begin{array}{ccc} P_{\mathrm{FA}} & = & \frac{\mathrm{number}\;\mathrm{of}\;\mathrm{false}\;\mathrm{detections}}{\mathrm{number}\;\mathrm{of}\;\mathrm{time}\;\mathrm{cells}-2},\\ P_{D} & = & \frac{\mathrm{number}\;\mathrm{of}\;\mathrm{true}\;\mathrm{detections}}{\mathrm{number}\;\mathrm{of}\;\mathrm{actual}\;\mathrm{targets}}. \end{array}$$

**Figure 8:**
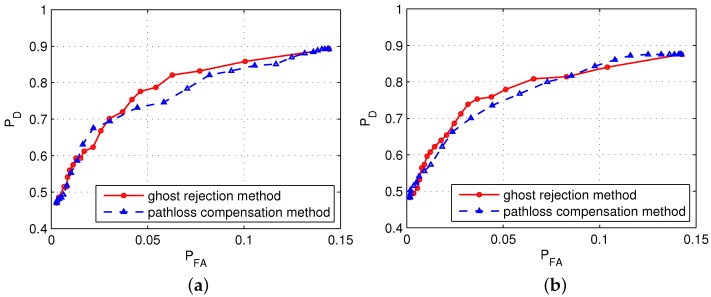

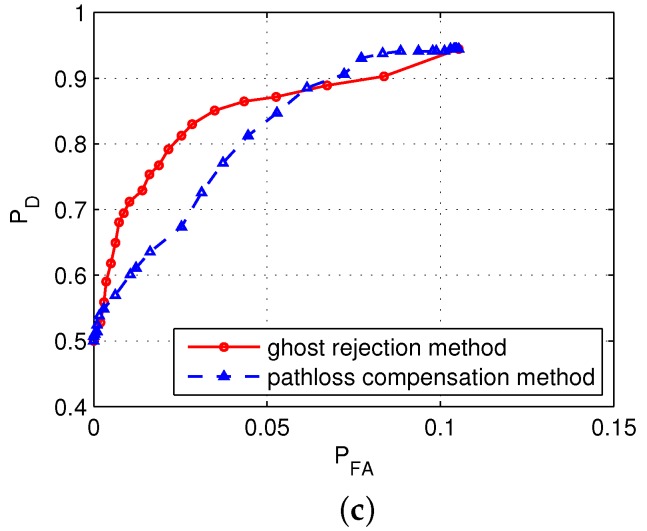
Receiver operating characteristic (RoC) curves for each scenario. (**a**) Scenario 1; (**b**) Scenario 2; and (**c**) Scenario 3.

Figure 8 shows a comparison of the receiver operating characteristic (RoC) curves obtained by applying the algorithm proposed in this paper and the path loss compensation method proposed in [10,11] in the three considered scenarios. In the figures, the results obtained via the former method shows a smaller $P_{\mathrm{FA}}$ for the given $P_{D}$ value than those obtained via the latter method. For example, in Scenario 3, when $P_{D}=0.81$, the value of $P_{\mathrm{FA}}$ is 0.025 if the former method is used, and 0.045 if the latter method is used, which are noticeably different values.

### 4.3. Test Results

**Figure 9:**
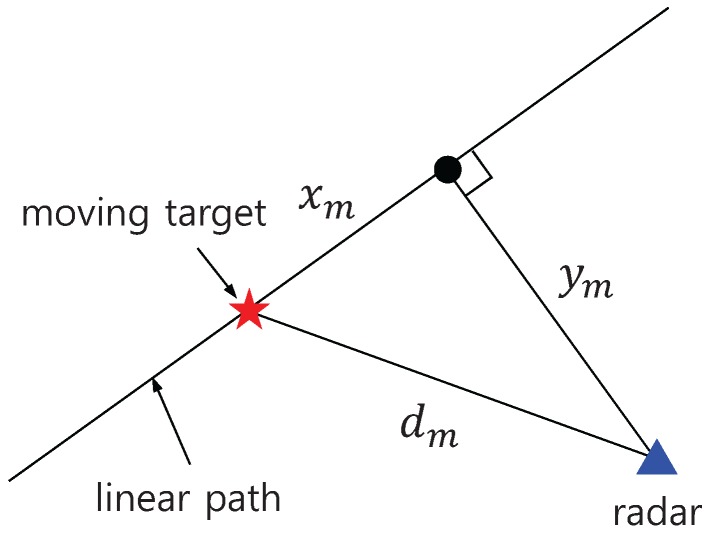
Test scenario.

We conducted 1D tracking in the three aforementioned scenarios by applying the ghost rejection algorithm proposed in the previous section. Figure 9 illustrates the measurement scenario introduced in Section 4.1. Parameter $d_{m}$ refers to the distance from the radar to the target in the $m^{\mathrm{th}}$ scan, $x_{m}$ indicates the range of the target, and $y_{m}$ denotes the distance to the straight line path along which the target moved. The multi-target tracking algorithm followed the conventional approach described in [12]. An extended Kalman filter with the following state model was used for the filtering and prediction process:

(14) $$\begin{array}{c} {\underline{x}}_{m+1}=F{\underline{x}}_{m}+{\underline{w}}_{m}, \end{array}$$

where

(15) $$\begin{array}{ccc} {\underline{x}}_{m} & = & {\left[x_{m}\;{\dot{x}}_{m}\;{\ddot{x}}_{m}\;y_{m}\right]}^{t}, \end{array}$$

(16) $$\begin{array}{ccc} F & = & \left[\begin{array}{cccc} 1\; & T_{s} & \frac{1}{2}T_{s}^{2} & 0\\ 0\; & 1 & T_{s} & 0\\ 0\; & 0 & 1 & 0\\ 0\; & 0 & 0 & 1 \end{array}\right]. \end{array}$$

The scan interval $T_{s}$ is 0.2 s, and the system noise ${\underline{w}}_{m}$ is a mean-zero Gaussian vector with the covariance matrixL:

(17) $$\begin{array}{c} K_{w}=\left[\begin{array}{cccc} 0 & 0 & 0 & 0\\ 0 & 0.1 & 0 & 0\\ 0 & 0 & 0.5 & 0\\ 0 & 0 & 0 & 0.01 \end{array}\right]. \end{array}$$

**Figure 10:**
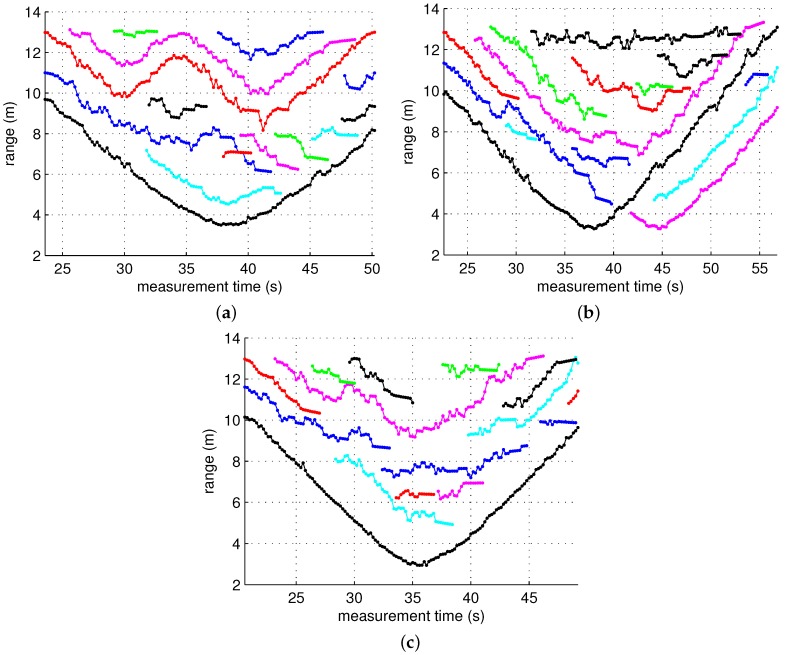
Tracking results for each scenario without ghost rejection. Separate tracks are indicated by different colors. (**a**) Scenario 1; (**b**) Scenario 2; and (**c**) Scenario 3.

**Figure 11:**
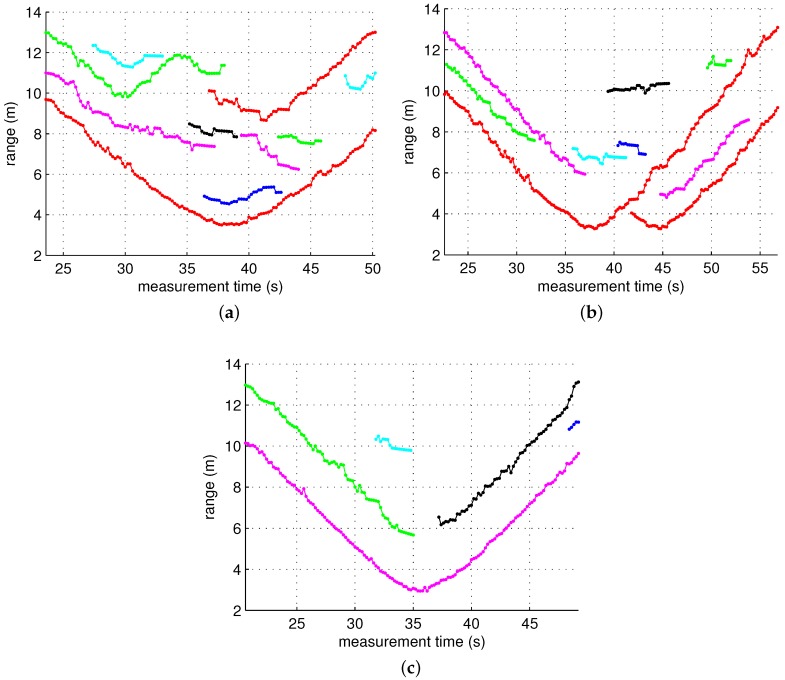
Tracking results for each scenario with ghost rejection. Separate tracks are indicated by different colors. (**a**) Scenario 1; (**b**) Scenario 2; and (**c**) Scenario 3.

The measurement model is given by:

(18) $$\begin{array}{c} d_{m}=\sqrt{x_{m}^{2}+y_{m}^{2}}+\epsilon_{m}, \end{array}$$

where the measurement noise $\epsilon_{m}$ is also Gaussian with a zero mean and a variance of 0.65. The widely used nearest neighbor method was employed for gating, and the gating threshold was chosen to be 0.87. The initial state is ${\underline{x}}_{0}={\left[x_{0}\;{\dot{x}}_{0}\;{\ddot{x}}_{0}\;y_{0}\right]}^{t}={\left[13\;-0.2\;0.01\;0.5\right]}^{t}$. Figure 10 and Figure 11 show the result of the tracking algorithm applied to three scenarios. In these figures, it is not easy to perfectly distinguish between true tracks and ghost tracks. However, the ghost tracks can be roughly identified by comparing them with the trajectory of the actual distance of the target shown in Figure 7, and it can be seen that the number of ghost tracks noticeably decreased when the ghost rejection algorithm was applied. For example, in Scenario 3, the number of ghost tracks decreased from eleven to two.

## 5. Conclusions

In this study, an indirect path that can occur in an indoor radar signal propagation was modeled statistically. We chose a widely used cluster model and modeled the ToA and the strength of the indirect path. Statistical modeling of an indirect path by a comprehensive measurement campaign is a unique contribution of this work. Applying the proposed model in 1D two-target tracking indicated that the number of ghost tracks is reduced effectively. This result is achieved by screening the candidate target obtained in the detection stage. If this is combined with the existing ghost rejection algorithm applied during the data association stage, we expect that additional performance enhancement will be possible.

## Figures and Tables

**Table 1:** Measurement environments. Measurements were taken in the lobbies, hallways, and lecture rooms of the university buildings.

Measurement Set	Location	Number of Scans
1	lecture room, 3rd floor, Main Library	187
2	lecture room, 3rd floor, Main Library	178
3	#313, 3rd floor, Newton Hall	287
4	lobby, 4th floor, Newton Hall	238
5	lobby, 4th floor, Newton Hall	275
6	lobby, 3rd floor, All Nations Hall	166
7	lobby, 3rd floor, All Nations Hall	227
8	hallway, 3rd floor, Nehemiah Hall	125
9	hallway, 3rd floor, Nehemiah Hall	111
10	hallway, 1st floor, Nehemiah Hall	108
11	hallway, 1st floor, Nehemiah Hall	80
12	lobby, 1st floor, Nehemiah Hall	143
13	lobby, 1st floor, Nehemiah Hall	168
	total	2293

**Table 2:** Fading parameters for the cluster model. The inter-arrival times of rays and clusters are modeled using exponential and gamma densities, respectively. The path strength follows lognormal fading.

Parameters	Symbol	Value
cluster arrival	*K*	8.4476
	*θ*	1.5510
ray arrival rate (1/ns)	*λ*	1.1521
cluster decay time constant (ns)	Γ	33.1268
ray decay time constant (ns)	*γ*	12.9967
standard deviation of cluster fading	$\sigma_{\mathrm{cluster}}$	1.6605
standard deviation of ray fading	$\sigma_{\mathrm{ray}}$	3.9038

## Appendix A

In this section, the conditional density $f_{\left.\underline{T\nu }\right|\underline{\mu }}\left(\left.{\underline{T}}^{\left(m\right)},{\underline{\nu }}^{\left(m\right)}\right|{\underline{\mu }}^{\left(m\right)}\right)$ shown in Equation (12) is evaluated. First, the $L_{m}$ row vectors of the observation matrix $R^{\left(m\right)}$ are partitioned into two groups: one associated with target 0, and the other associated with target 1. Let the observation matrix created by the target $q\left(q=0,1\right)$ be $R_{q}^{\left(m\right)}$. Then,

(A1) $$R_{q}^{\left(m\right)}=\left[{\underline{T}}_{q}^{\left(m\right)},\;{\underline{\nu }}_{q}^{\left(m\right)}\right]={\left[T_{n,q}^{\left(m\right)},\nu_{n,q}^{\left(m\right)}\right]}_{L_{m,q}\times 2},\;0\leq n\leq L_{m,q}-1,$$

where $\left(T_{n,q}^{\left(m\right)},\nu_{n,q}^{\left(m\right)}\right)\in \left\{\left.\left(T_{l}^{\left(m\right)},\nu_{l}^{\left(m\right)}\right)\right|\mu_{l}^{\left(m\right)}=q\right\}$ and $T_{0,q}^{\left(m\right)}<T_{1,q}^{\left(m\right)}<\cdots <T_{L_{m,q}-1,q}^{\left(m\right)}$. By calibrating the observation matrix, we define ${\tilde{R}}_{q}^{\left(m\right)}$ as:

(A2) $${\tilde{R}}_{q}^{\left(m\right)}=\left[{\tilde{\underline{T}}}_{q}^{\left(m\right)},\;{\tilde{\underline{\nu }}}_{q}^{\left(m\right)}\right]={\left[{\tilde{T}}_{n,q}^{\left(m\right)},{\tilde{\nu }}_{n,q}^{\left(m\right)}\right]}_{L_{m,q}\times 2},\;0\leq n\leq L_{m,q}-1,$$

where ${\tilde{T}}_{n,q}^{\left(m\right)}=T_{n,q}^{\left(m\right)}-T_{0,q}^{\left(m\right)}$ and ${\tilde{\nu }}_{n,q}^{\left(m\right)}=\nu_{n,q}^{\left(m\right)}-\phi_{q}^{\left(m\right)}$. That is, the ToA of each cluster is calibrated to become $T_{0,q}^{\left(m\right)}=0$, and the cluster energy is scaled by the parameter $\phi_{q}^{\left(m\right)}$ selected to have the minimum squared error. Here, the parameter $\phi_{q}^{\left(m\right)}$ can be defined as:

(A3) $$\begin{array}{c} \phi_{q}^{\left(m\right)}=\arg\min_{\phi }\left[\sum_{n=0}^{L_{m,q}}{\left[\nu_{n,q}^{\left(m\right)}-\phi +\frac{10{\tilde{T}}_{n,q}^{\left(m\right)}}{\Gamma \ln10}\right]}^{2}\right]. \end{array}$$

Since parameters ${\left\{{\tilde{T}}_{n,q}^{\left(m\right)},{\tilde{\nu }}_{n,q}^{\left(m\right)}\right\}}_{n=0}^{L_{m,q}-1}$ are all independent, the following condition is satisfied:

(A4) $$\begin{array}{c} f_{\left.\underline{T\nu }\right|\underline{\mu }}\left(\left.{\underline{T}}^{\left(m\right)},{\underline{\nu }}^{\left(m\right)}\right|{\underline{\mu }}^{\left(m\right)}\right)=f_{\left.\underline{\tilde{T}\tilde{\nu }}\right|\tilde{\underline{\mu }}}\left(\left.{\tilde{\underline{T}}}^{\left(m\right)},{\tilde{\underline{\nu }}}^{\left(m\right)}\right|{\tilde{\underline{\mu }}}^{\left(m\right)}\right)\\ \;=\prod_{q=0}^{1}\prod_{n=1}^{L_{m,q}}f_{\delta }\left({\tilde{T}}_{n,q}^{\left(m\right)}-{\tilde{T}}_{n-1,q}^{\left(m\right)}\right)\cdot f_{\left.\tilde{\nu }\right|\tilde{T}}\left(\left.{\tilde{\nu }}_{n,q}^{\left(m\right)}\right|{\tilde{T}}_{n,q}^{\left(m\right)}\right), \end{array}$$

where the marginal density $f_{\delta }\left(\delta \right)$ is given by Equation (5), and the conditional density of $\tilde{\nu }$ is given by:

(A5) $$\begin{array}{c} f_{\left.\tilde{\nu }\right|\tilde{T}}\left(\left.\tilde{\nu }\right|\tilde{T}\right)=\frac{1}{\sqrt{2\pi }\sigma_{\mathrm{cluster}}}\exp\left[-\frac{{\left(\tilde{\nu }+\frac{10\tilde{T}}{\Gamma \ln10}\right)}^{2}}{2\sigma_{\mathrm{cluster}}^{2}}\right]. \end{array}$$
